# Visualisation of Multiple Tight Junctional Complexes in Human Airway Epithelial Cells

**DOI:** 10.1186/s12575-018-0070-0

**Published:** 2018-02-01

**Authors:** Alysia G. Buckley, Kevin Looi, Thomas Iosifidis, Kak-Ming Ling, Erika N. Sutanto, Kelly M. Martinovich, Elizabeth Kicic-Starcevich, Luke W. Garratt, Nicole C. Shaw, Francis J. Lannigan, Alexander N. Larcombe, Graeme Zosky, Darryl A. Knight, Paul J. Rigby, Anthony Kicic, Stephen M. Stick

**Affiliations:** 1 Centre of Microscopy, Characterisation and Analysis, The University of Western Australia, Crawley, Western Australia 6009 Australia; 2 School of Paediatrics and Child Health, The University of Western Australia, Nedlands, Western Australia 6009 Australia; 3 Centre for Cell Therapy and Regenerative Medicine, School of Medicine and Pharmacology, The University of Western Australia, Nedlands, Western Australia 6009 Australia; 4 Telethon Kids Institute, Centre for Health Research, The University of Western Australia, Crawley, Western Australia 6009 Australia; 5 Department of Respiratory Medicine, Princess Margaret Hospital for Children, Perth, Western Australia 6001 Australia; 6 School of Medicine, Notre Dame University, Fremantle, Western Australia 6160 Australia; 7 School of Medicine, Faculty of Health, University of Tasmania, Hobart, Tasmania 7000 Australia; 8 School of Biomedical Sciences and Pharmacy, University of Newcastle, Callaghan, New South Wales Australia; 9 Priority Research Centre for Asthma and Respiratory Disease, Hunter Medical Research Institute, Newcastle, New South Wales Australia; 10 Department of Anesthesiology, Pharmacology and Therapeutics, University of British Columbia, Vancouver, Canada; 11 School of Public Health, Curtin University, Bentley, Western Australia 6102 Australia; 12 Telethon Kids Institute, Subiaco, Perth, Western Australia 6008 Australia

**Keywords:** Tight junctions, Confocal microscopy, Fixation, Airway epithelial cells, Air liquid interface

## Abstract

**Background:**

Apically located tight junctions in airway epithelium perform a fundamental role in controlling macromolecule migration through paracellular spaces. Alterations in their expression may lead to disruptions in barrier integrity, which subsequently facilitates entry of potential bacterial and other pathogens into the host. Furthermore, there is emerging evidence that the barrier integrity of the airway in certain airway inflammatory diseases may be altered. However, there is little consensus on the way this is assessed and measured and the type of cells used to achieve this.

**Methods:**

Here, we assessed four fixation methods including; (i) 4% (*v*/v) paraformaldehyde; (ii) 100% methanol; (iii) acetone or; (iv) 1:1 methanol: acetone. Pre-extraction with Triton X-100 was also performed and assessed on cells prior to fixation with either methanol or paraformaldehyde. Cells were also permeabilized with 0.1% (v/v) Saponin in 1× TBS following fixation and subsequently stained for tight junction proteins. Confocal microscopy was then used to visualise, compare and evaluate staining intensity of the tight junctional complexes in order to determine a standardised workflow of reproducible staining.

**Results:**

Positive staining was observed following methanol fixation for claudin-1 and ZO-1 tight junction proteins but no staining was detected for occludin in 16HBE14o- cells. Combinatorial fixation with methanol and acetone also produced consistent positive staining for both occludin and ZO-1 tight junction proteins in these cells. When assessed using primary cells cultured at air-liquid interface, similar positive staining for claudin-1 and ZO-1 was observed following methanol fixation, while similar positive staining for occludin and ZO-1 was observed following the same combinatorial fixation with methanol and acetone.

**Conclusions:**

The present study demonstrates the importance of a personalised approach to optimise staining for the visualisation of different tight junction proteins. Of significance, the workflow, once optimised, can readily be translated into primary airway epithelial cell air-liquid interface cultures where it can be used to assess barrier integrity in chronic lung diseases.

## Background

The airway epithelial layer remains the frontline of defence against pathogens, aeroallergens and noxious gases by establishing and maintaining a physical barrier. The integrity of this layer is typically maintained by the presence of a range of junctional complexes including: tight junctions; adherens junctions; and desmosomes [1–4]. Apically located tight junctions perform a fundamental role in regulating solute transport across the airway epithelium [5] by restricting macromolecule migration through paracellular spaces [6–9]. Several families of proteins have been identified to form tight junctions between adjacent cells including the occludin and claudin families. These proteins contain four transmembrane domains with two extracellular loops, where the extracellular loops fuse with their counterpart on adjacent cells [10] resulting in a belt-like structure around the apical surface of airway epithelial cells [4, 5, 11, 12]. In association with the transmembrane tight junction proteins is the intracellular protein zona occludens-1 (ZO-1) [10] which act by anchoring the tight junction proteins to the cytoskeleton [13].

Studies have observed that decreases in ZO-1, claudin-1 and occludin organisation within the cell membrane leads to disruption of barrier function in epithelial cells, thereby allowing entry of bacteria and other pathogens into the host [6–9, 14, 15]. Some evidence also suggests that alteration of adherens junctions can also facilitate the entry of pathogens into the host [6, 14]. Recent investigations suggest that epithelial barrier integrity may be dysfunctional in airway diseases such as asthma, where decreased tight junctional complexes and increased layer permeability have been observed [3, 16, 17]. Tight junction proteins ZO-1 and occludin have also been shown to have lower expression and a disorganised structure in asthmatic epithelium, when compared to non-asthmatic epithelium, resulting in reduced barrier function [15, 16].

Tight junction integrity has typically been assessed using Transepithelial Electrical Resistance (TEER) [18]. Higher resistance measurements are typically observed in confluent polarised cultures with intact junctional complexes since ions cannot pass across the epithelial cellular layer into basal compartments [19]. Conversely, low TEER values are a consequence of increased ion transport across the epithelial layer, indicative of increased permeability resulting from incomplete tight junctions [15, 19, 20]. Despite these measurements providing insight into the global changes, they fail to provide insight into localised changes that may be occurring between cells. Thus, confocal microscopy provides a valuable tool for the visualisation and assessment of local protein changes and interactions, and may also be used to corroborate TEER measurements. Here, we optimised and established a methodology for epithelial tissue fixation for the immunocytochemical analysis of tight junctions (ZO-1, claudin-1 and occludin), initially in a representative airway epithelial cell line (16HBE14o-), followed by corroboration in primary airway epithelial cultures grown at air-liquid interface (ALI).

## Methods

### Reagents

The culture reagents Modified Eagle’s Medium (MEM), Penicillin/Streptomycin, L-Glutamine, Foetal Calf Serum (FCS) and Normal Goat Serum (NGS) were purchased from Life Technologies (CA, USA). Triton X-100, trizma base, sodium chloride, bovine serum albumin (BSA) and fibronectin were purchased from Sigma Aldrich (MO, USA). Collagen IV was purchased from BD Biosciences (New Jersey, USA).

### Antibodies

For immunocytochemistry, the following antibodies were used: Claudin-1 (polyclonal), Occludin (monoclonal, clone OC-3F10), ZO-1 (monoclonal, clone ZO1-1A12, and polyclonal), AlexaFluor 488 (Goat anti-Mouse and Goat anti-Rabbit) and AlexaFluor 568 (Goat anti-Mouse and Goat anti-Rabbit). These antibodies were purchased from Life Technologies (CA, USA). Hoechst 33,342 was purchased from Sigma Aldrich (MO, USA).

### Cell Culture and Maintenance

16HBE14o- cells, a SV-40 transformed bronchial epithelial cell line, were kindly provided by Dr. Dieter Gruenet (University of California, San Francisco, USA). Cells were cultured in MEM containing 10% (*v*/v) FCS, 100 U/mL (v/v) Penicillin/Streptomycin and 1% (v/v) L-Glutamine in a 37 °C, 5% CO_2_ incubator. For experiments, cells were seeded at a density of 10,000 cells/coverslip on glass coverslips previously coated with 10 μg/mL fibronectin, 30 μg/mL collagen I and 100 μg/mL BSA. Cells were maintained under standard culture conditions until 100% confluency over the coverslips was achieved. Cultures were then continued for a further 3 days before being fixed for subsequent immunocytochemical analysis to ensure complete generation of tight junction proteins.

### Establishment of ALI Cultures

Primary airway epithelial cells (AECs) were obtained from children admitted for elective surgery for non-respiratory related conditions [21–23] and de-identified prior to downstream analysis. Primary AECs were then grown on 6.5-mm Transwell-Clear inserts 0.4 μm pore size (Corning, NY, USA) pre-coated with 30 μg/mL human placental collagen type I, which has been previously demonstrated to support AEC growth [24]. Cells were grown under submerged conditions in Bronchial-Air Liquid Interface (B-ALI™, Lonza, MD, USA) growth media until confluent. To differentiate into ciliated pseudostratified AECs, media was removed from the apical side and this was considered Day 0 of ALI culture and the start of the experimental period. Cells were then grown in B-ALI™ differentiation media, added to the basolateral side every alternate day and the apical side washed with tissue-culture sterile 1X PBS weekly. Cultures were grown for 28 days at ALI to ensure maximal differentiation as assessed by the presence of beating cilia as well as mucus production, as evident by mucus build-up on the apical side of the cultures.

### Fixation

This study sought to investigate various fixation methods suitable for the reproducible staining of epithelial airway cells. All treatments were repeated in triplicate. All fixation combinations can be found in Table 1.

**Table 1:** Fixative combinations used in this study. All fixative combinations were performed in triplicate on 16HBE14o- cultured cells. Immunocytochemistry was performed on cells as detailed

Pre-extraction	Fixation	Permeabilization
–	4% Paraformaldehyde	–
0.2% Triton X-100	4% Paraformaldehyde	–
–	4% Paraformaldehyde	0.1% Saponin
–	4% Paraformaldehyde + Acetone	–
–	100% Methanol	–
0.2% Triton X-100	100% Methanol	–
–	100% Methanol	0.1% Saponin
–	1:1 Methanol:Acetone	–
–	100% Acetone	–

#### Paraformaldehyde

Cells were fixed using 4% (v/v) paraformaldehyde in 71 mM Tris Buffered Saline (TBS), pH 7.4, at room temperature (RT) for 15 min, followed by washing with TBS for 30 min at RT, replacing wash TBS every 5 min. Cells were then stored in TBS at 4 °C until required.

#### Methanol, Acetone, Methanol: Acetone

Fixation with coagulant fixatives was performed using either ice cold 100% methanol, acetone or 1:1 methanol: acetone. Cells were fixed at − 20 °C for 10 min, followed by washing with TBS for 30 min at RT, replacing wash TBS every 5 min. Cells were then stored in TBS at 4 °C until required.

#### Triton X-100 Pre-extraction

Cells were incubated with 0.2% (*v*/v) Triton X-100 in 1× TBS on ice for 10 min, followed by gentle washing with TBS for 30 min at RT, replacing wash TBS every 5 min. Fixation following pre-extraction was performed with either methanol or paraformaldehyde as described above. Cells were then stored in TBS at 4 °C until required.

### Permeabilization

Following fixation, permeabilization was performed on a number of samples. Here, cells were treated with 0.1% (v/v) Saponin in 1× TBS and incubated at RT for 10 min. Cells were then washed with 1× TBS for 30 min at RT, replacing wash TBS every 5 min. Cells were then stored in TBS at 4 °C until required.

### Blocking Solution

To minimise non-specific binding of primary and secondary antibodies in samples, blocking solution containing 10% (v/v) NGS, 10% (v/v) FCS and 1% (v/v) BSA in 1× TBS was incubated on cells for 30 min at RT. For paraformaldehyde fixed samples, 0.2% (v/v) Triton X-100 was included in the blocking solution. In addition, all antibodies were diluted in the blocking solution outlined above.

### Immunocytochemistry

Primary antibodies were incubated on cells for 1 h at RT, followed by washing with 1× TBS at RT every 10 min for 1 h. Secondary antibody incubation and wash was performed as per the primary antibody incubation step. Cells were also incubated with Hoechst (2.5 μg/mL) for 5 min at RT during the final wash step to stain for nuclei. All coverslips were mounted with mounting medium containing 19 mM polyvinyl alcohol (PVA, Sigma Aldrich, MO, USA), 45 mM Trizma Base (Sigma Aldrich, MO, USA), 45 mM NaH_2_PO_4_.2H_2_O, 27% (v/v) glycerol (Sigma Aldrich, MO, USA), and 4.9 mM chlorobutanol (Sigma Aldrich, MO, USA). Negative control samples were included to determine the level of non-specific binding of secondary antibodies to the tissue.

### Confocal Microscopy

Treated and control samples were imaged using a Nikon A1 inverted confocal microscope (Nikon, Japan), with a Nikon Plan Apo VC 60× NA 1.4 oil immersion objective (Nikon, Japan) and NIS-AR Elements software (v4.2.22, Nikon, Japan). Individual channels were captured sequentially, where a 405 nm laser was used for Hoechst 33,342 with collection through a 450/50 bandpass filter, AF488 excited using a 488 nm laser with collection through 525/50, and AF568 excited with a 561 nm laser and collected through a 585/50 bandpass filter. Z-stack images with step size of 0.5 μm were collected with a pinhole of 35.8 μm (1.2 A.U. for 488 nm laser), where the top and bottom of the stacks were determined visually.

## Results

**Fig. 1:**
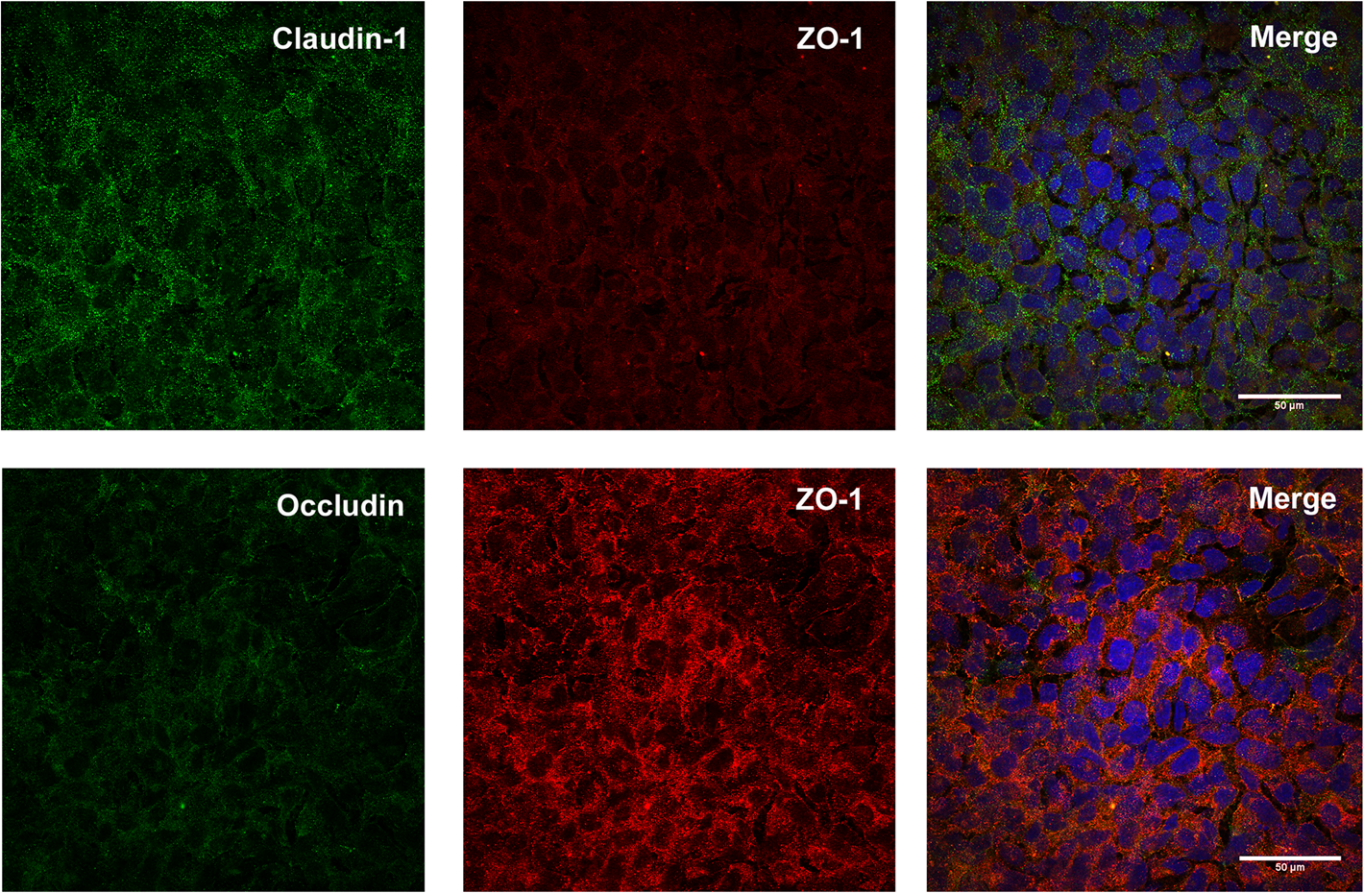
Paraformaldehyde fixation (4%) of 16HBE14o- cells in culture. The top row of panels show absence of specific staining for claudin-1 (Green) and ZO-1 (Red). The bottom row of panels show co-staining of occludin with ZO-1. Merged images showed nuclei staining with Hoechst (blue)

To determine the extent of tight junction formation in epithelial cells we examined the effect of various fixatives on the epithelial cell line 16HBE14o- (Table 2). Initial experiments did not produce staining for any fixation combinations where fibronectin/collagen coating of coverslips was omitted (data not shown). Coverslip coating was used following these preliminary experiments to aid adherence and cell growth [25]. Image analysis of paraformaldehyde fixed cells, with a Triton X-100 permeabilization step, showed no specific staining of tight junction complexes ZO-1, occludin or claudin-1 (Fig. 1). Saponin was used following paraformaldehyde fixation as an alternative permeabilization agent to Triton X-100. Data generated showed that use of saponin slightly increased junctional staining post paraformaldehyde fixation for ZO-1, but positive staining was highly variable within samples (data not shown). Occludin and claudin-1 staining was absent following paraformaldehyde-saponin fixation and permeabilization. Pre-extraction with 0.2% Triton X-100 on ice, followed by fixation, was also tested. However, following the pre-extraction treatment, all cells lost attachment to the coverslip and immunocytochemistry was not performed (data not shown). Using a combinatorial approach of paraformaldehyde fixation, followed by acetone fixation to increase permeabilization, also failed to yield positive staining for ZO-1, occludin or claudin-1 (data not shown).

**Table 2:** Qualitative assessment of fluorescent staining of tight junction antibodies, where: - indicates negative staining; + indicates weak staining with no consistent structure; ++ indicates moderate staining of tight junctions, with some structure present; +++ indicates strong staining, with consistent structures present

Fixation	Claudin-1	Occludin	ZO-1
4% Paraformaldehyde	–	–	–
0.2% Triton Pre-extraction + 4% Paraformaldehyde	–	–	–
4% Paraformaldehyde + 0.1% Saponin	–	–	+
4% Paraformaldehyde + Acetone	–	–	–
Methanol	+++	–	+++
0.2% Triton Pre-extraction + Methanol	–	–	–
Methanol + 0.1% Saponin	+	–	+
Methanol + Acetone	–	+++	+++
Acetone	–	–	–

**Fig. 2:**
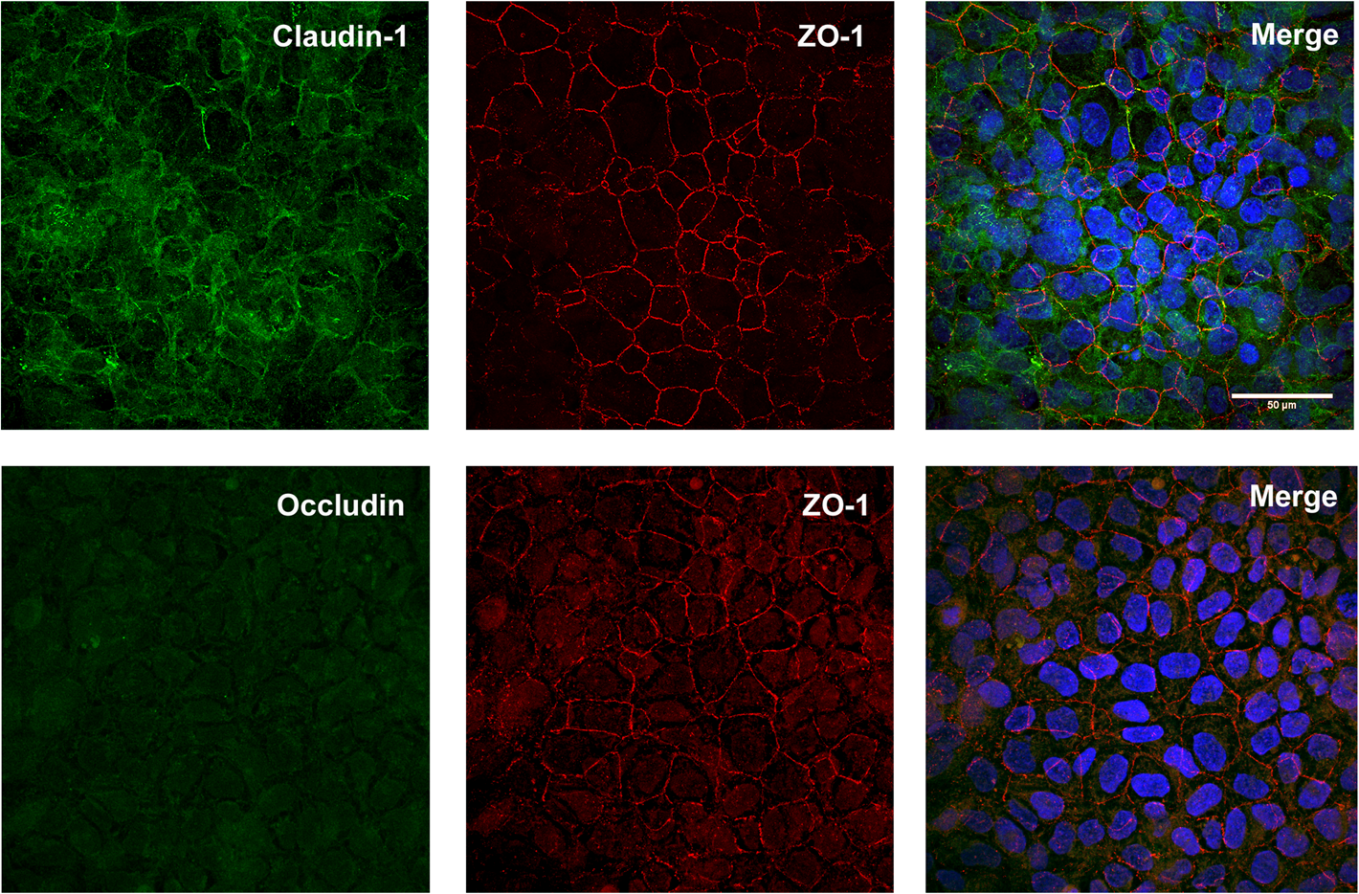
Methanol fixation of 16HBE14o- cells in culture. The top row of panels show claudin-1 co-stained with ZO-1. The bottom row of panels show the destruction of occludin staining using methanol as the fixative. Merged images showed nuclei staining with Hoechst (blue)

**Fig. 3:**
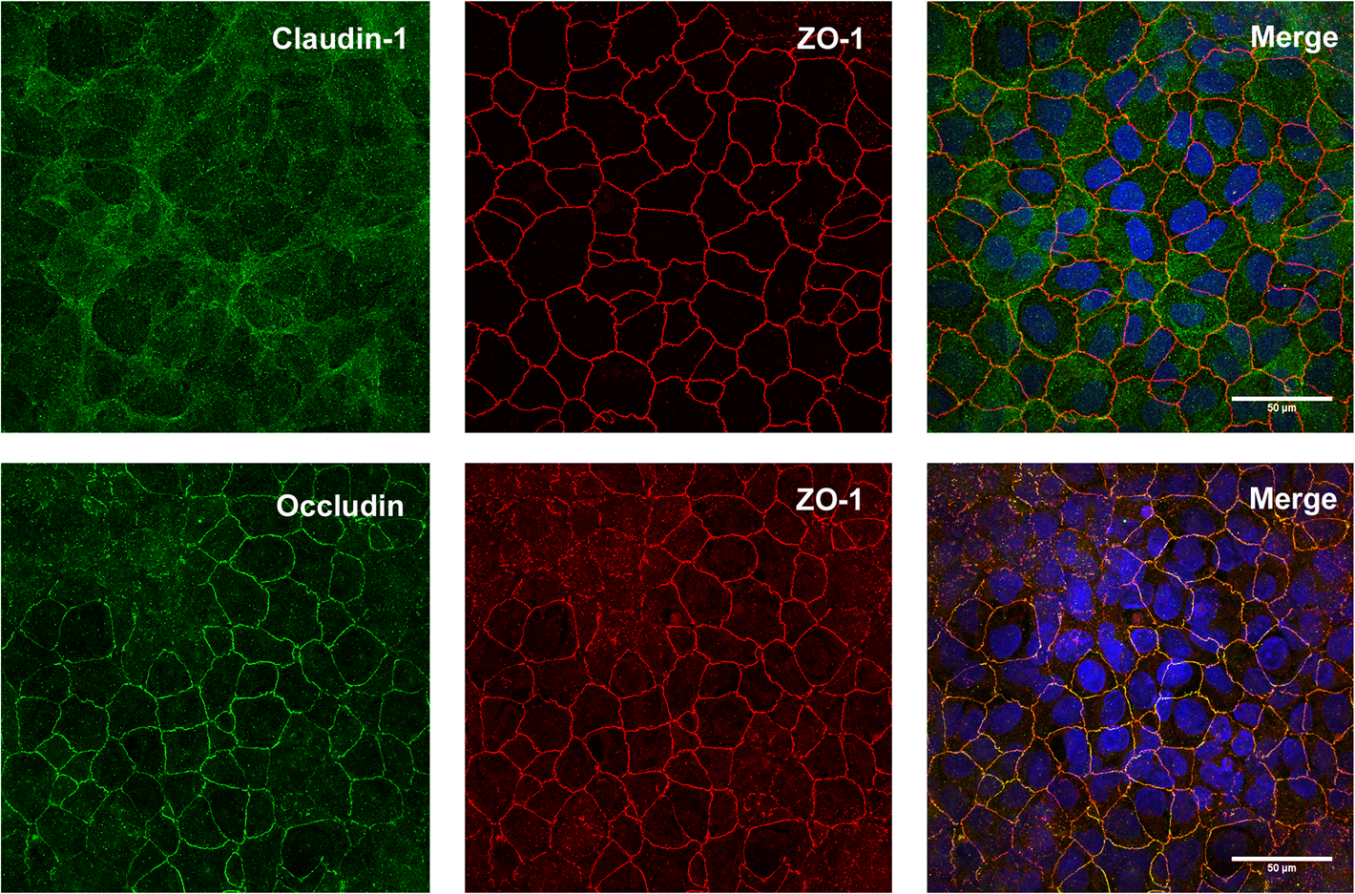
Methanol-acetone (1:1) fixation of 16HBE14o- cells in culture. The top row of panels show the absence of claudin-1 staining, whilst ZO-1 staining is clearly visible. The bottom row of panels show co-staining of occludin with ZO-1 using methanol-acetone as the fixative. Merged images showed nuclei staining with Hoechst (blue)

Coagulative fixation methods were also tested to determine epitope accessibility. Positive staining was observed following methanol fixation for ZO-1 and claudin-1 tight junction proteins, but no staining was detected for occludin (Fig. 2). Fixation with acetone failed to expose any positive staining for ZO-1, occludin or claudin-1 in the samples (data not shown). Permeabilization with saponin following methanol fixation was unsuccessful in producing tight junction staining for any of the antibodies assessed (data not shown). Combinatorial coagulative fixation produced consistent positive staining for both ZO-1 and occludin. However, no claudin-1 staining was observed (Fig. 3). No fluorescence was observed in negative controls for all fixation methods, where positive sample settings were used (data not shown).

**Fig. 4:**
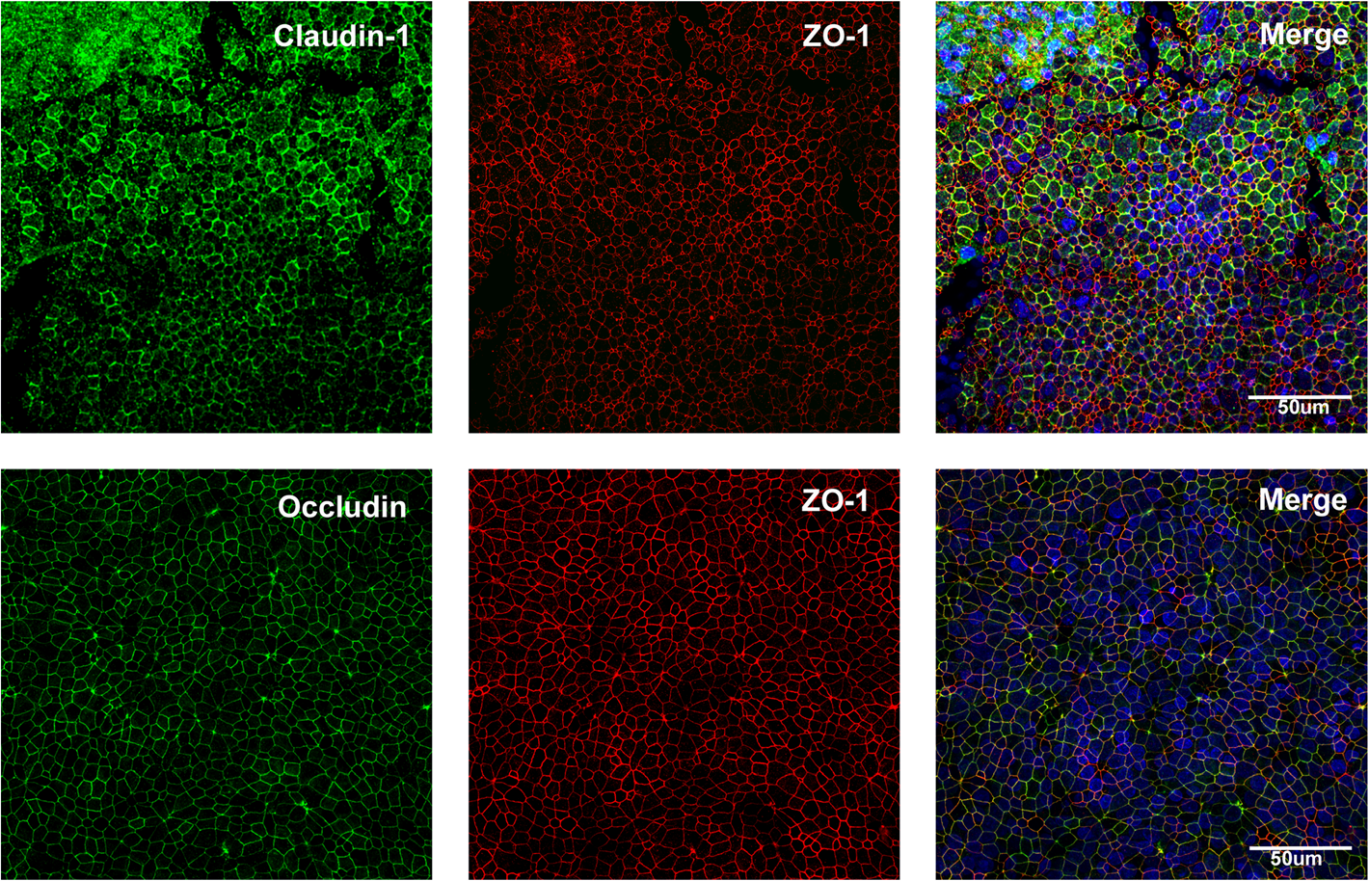
Methanol-acetone fixation of primary airway epithelial cells (AEC) grown at air liquid interface (ALI). The top row of panels show positive staining for both claudin-1 and ZO-1. The bottom row of panels show positive co-staining of occludin and ZO-1 in primary AECs grown at ALI. Merged images showed nuclei staining with Hoechst (blue)

Following optimisation of the staining protocol, staining on primary cells cultured to ALI was performed to verify compatibility between the transformed cell line and primary cells. Fixation of ALI culture with methanol showed positive staining for both claudin-1 and ZO-1, as seen with the 16HBE14o- cell line, whilst fixation with 1:1 methanol: acetone produced positive staining for occludin and ZO-1 (Fig. 4).

## Discussion

In the current study, we found that fixation of 16HBE14o- cells for the tight junction proteins ZO-1, claudin-1 and occludin require different fixation protocols for reliable staining patterns. For consistent staining of claudin-1, fixation with ice cold methanol was required, whilst occludin needed a combinatorial approach of methanol: acetone. We found that staining for ZO-1 could be positively identified using both fixation approaches, and as such, could be used as a counter stain for both claudin-1 and occludin. Furthermore, we found that staining patterns in cell line 16HEB14o- was congruent with primary airway epithelial cells grown in ALI. This consistency in staining pattern reinforces their usefulness as a substitute for protocol optimization, as access to paediatric primary epithelial cells is often limited.

Methodologies to perform reproducible immunocytochemistry for tight junction proteins (ZO-1, occludin and claudin-1) in epithelial derived cells to date are inconsistent and, at times, conflicting [6, 26, 27]. Particularly for epithelial cells, the fixation method must be carefully chosen to ensure optimal staining for the antigens of interest [28, 29]. Routine histological fixatives are often used without thought as to why one fixative may be better suited for a particular antigenic epitope than another.

The most common types of fixatives used in immunocytochemistry fall into two categories: (1) non-coagulative or cross-linking and (2) coagulative fixatives. The cross-linking family include formaldehyde and glutaraldehyde [29]. These fixatives transform the cytosol into an insoluble gel by the formation of methylene bridges between proteins, which halt autolysis and harden tissue [30, 31]. Fixation via this method may alter some of the tertiary protein structure within the tissue, but generally maintains secondary protein structures [29, 31–33]. Absence of claudin-1 and occludin antibody labelling using this fixation method in our laboratory may be due to the location of the proteins within the cell membrane, preventing sufficient access of the antibody to the epitope. Whilst the ZO-1 protein is not located within the cellular membrane, the negative staining following paraformaldehyde fixation may also be attributable to the restricted epitope access due to cross-linked adjacent proteins, or steric hindrance.

As the cross-linking fixatives change cytoplasm into an insoluble gel, permeabilization steps may be required for immunocytochemical analysis of intracellular components [32, 34, 35]. Surfactants and non-ionic detergents, such as saponin and Triton X-100 respectively, are commonly used in immunocytochemistry for the purpose of increasing cellular permeability [35–38]. Solubilisation of lipid components non-specifically by Triton X-100, or specific cholesterol removal by saponin, facilitates antibody access to intracellular compartments and epitopes without changing the cells’ ultrastructural integrity [28]. Saponin might be expected to increase epitope exposure for ZO-1, occludin or claudin-1. However, in our samples, permeabilization with saponin did not alter antibody staining of occludin or claudin-1 tight junction proteins, with occasional variable staining for ZO-1. This negligible staining following the use of surfactants may be due to the epitope for claudin-1 and occludin being located in a position that is not altered by the removal of, nor coupled to, lipids or cholesterol.

Pre-extraction with Triton X-100, followed by fixation with paraformaldehyde, has been suggested to remove some background staining in tissues, as some soluble components within the cell are removed prior to fixation [29, 34]. As such, this should provide greater access for antibodies to bind to epitopes of interest, as the lipids are removed in a non-selective manner. However, following exposure of confluent 16HBE14o- cells to Triton X-100, all cells appeared to lose attachment to the extracellular matrix (ECM). There are also suggestions that paraformaldehyde is unable to sufficiently cross-link proteins in situ [39], although other studies suggest that formaldehyde is only released from tissues following years of washing tissues in water, and cross-linking bonds cannot be broken by urea [40]. In our study, it is likely that the epitopes of interest are insufficiently exposed via the cross-linking fixation and permeabilization methods commonly employed.

Coagulant fixatives are also commonly used to fix tissue for immunocytochemistry. This family includes alcohols such as ethanol and methanol, as well as acetone [33]. Fixation of our samples with coagulant fixatives produced varied results. The use of methanol fixation revealed positive staining for ZO-1, but occludin staining was absent. Alcohol fixatives simultaneously fix and permeabilize cells, by extracting phospholipids and precipitating proteins in tissue [41]. They are frequently used for observing cellular cytoskeletal elements, as shown with the positive ZO-1 tight junction staining. The coagulant fixatives displace water molecules from proteinaceous materials, thereby breaking hydrogen bonds [42]. Alterations of hydrogen bonds can change the tertiary structure of proteins but does not alter the amino acid sequence of the epitope [42]. This can result in exposure of epitopes which were previously buried within the protein, thereby allowing antibody access and binding [42]. This alteration of protein tertiary structure protein may not have been sufficient to unmask the occludin epitope, and as such, further investigation was required.

Acetone is another coagulative fixative with strong lipid removal activity, particularly triglycerides and sterols [43]. In our samples, fixation with acetone failed to produce positive staining for any tight junction proteins. As acetone is a stronger organic solvent than alcohol, cell membrane loss can be observed following cellular fixation [31, 43]. To change the epitope exposure, without complete loss of cellular membranes, a fixative of 1:1 methanol: acetone was performed. Staining following dual fixation showed positive fluorescence for ZO-1 and occludin proteins, however claudin-1 staining was destroyed. It is plausible that the extra denaturation required for occludin antibody access results in masking of the claudin-1 epitope.

The optimized fixation protocol was then repeated on primary airway epithelial cell culture samples derived from healthy participants, where cells had successfully reached a differentiated state when grown under ALI conditions. Fixation of the cultures with methanol yielded positive staining with claudin-1 and ZO-1, whilst methanol: acetone fixation yielded positive staining for occludin and ZO-1, reproducing the findings seen with the 16HBE14o^−^ cultures. However, it should be noted that there were subtle differences in the staining intensity as well as the pattern of staining, suggesting that the final interpretation of staining should be restricted to primary cultures and not with the surrogate optimisation model.

## Conclusions

**Fig. 5:**
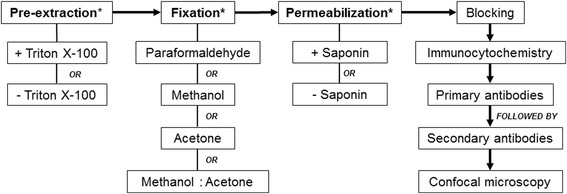
Schematic representation of the workflow required for the visualization of tight junctional complexes in airway epithelial cells. * denotes key points within the workflow which requires specialized optimization

In conclusion, this study successfully established a methodological workflow (Fig. 5) using confocal microscopy to compare and evaluate staining expression levels of multiple tight junction complexes in the human airway. Via the workflow, we established that there was no universal methodological approach appropriate for staining and visualising all tight junction proteins investigated. However, we identified key points within the methodological workflow which after specialised optimisation lead to subsequent visualisation of each tight junction protein. Finally, we successfully demonstrated the reproducibility and translation of the workflow in primary AEC ALI cultures, indicating the adaptability of this method in other cell types. Of significance, this workflow can now be used to visualise epithelial tight junctions and assess barrier integrity in established cell cultures derived from chronic airway diseases including cystic fibrosis, chronic obstructive pulmonary disorder and asthma.

## Abbreviations

AEC: Airway epithelial cell
ALI: Air-liquid interface
B-ALI: Bronchial Air-Liquid Interface
BSA: Bovine Serum Albumin
FCS: Foetal Calf Serum
MEM: Minimum Essential Medium
NGS: Normal Goat Serum
pAECs: Primary airway epithelial cells
PBS: Phosphate Buffered Saline
PVA: Polyvinyl Alcohol
RT: Room Temperature
TBS: Tris Buffered Saline
TEER: Transepithelial Electrical Resistance
ZO-1: Zona Occludens-1
